# Association of insulin-like growth factor-binding protein-3 with radiotherapy response and prognosis of esophageal squamous cell carcinoma

**DOI:** 10.1186/s40880-015-0046-2

**Published:** 2015-09-14

**Authors:** Li-Ling Luo, Lei Zhao, Mian Xi, Li-Ru He, Jing-Xian Shen, Qiao-Qiao Li, Shi-Liang Liu, Peng Zhang, Dan Xie, Meng-Zhong Liu

**Affiliations:** Sun Yat-sen University Cancer Center, State Key Laboratory of Oncology in South China, Collaborative Innovation Center for Cancer Medicine, Guangzhou, 510060 Guangdong P.R. China; Department of Radiation Oncology, Sun Yat-sen University Cancer Center, Guangzhou, 510060 Guangdong P.R. China; Medical Imaging and Interventional Center, Sun Yat-sen University Cancer Center, Guangzhou, 510060 Guangdong P.R. China; Collaborative Innovation Center for Cancer Medicine, Guangzhou, 510060 Guangdong P.R. China

**Keywords:** Esophageal squamous cell carcinoma, Insulin-like growth factor-binding protein-3, Immunohistochemistry, Radiotherapy response, Prognosis

## Abstract

**Background:**

Insulin-like growth factor-binding protein-3 (IGFBP-3) is suggested to predict the radiosensitivity and/or prognosis of patients with esophageal squamous cell carcinoma (ESCC). The present study was designed to investigate the clinical and prognostic effects of IGFBP-3 on ESCC.

**Methods:**

IGFBP-3 was detected by immunohistochemistry in paraffin-embedded tissues from 70 ESCC patients treated with radiotherapy alone and further examined by western blotting analysis in 10 pairs of fresh ESCC tissues and adjacent non-malignant esophageal specimens. Receiver operating characteristic (ROC) analysis was used to determine cut-off scores for tumor positivity and to evaluate patient survival status. The χ^2^ test was performed to analyze the association of IGFBP-3 expression with clinical characteristics and radiotherapy response. Associations between prognostic outcomes and IGFBP-3 expression were investigated using Kaplan–Meier analysis and the Cox proportional hazards model.

**Results:**

The threshold for IGFBP-3 positivity was set to greater than 65% [area under the ROC curve (AUC) = 0.690, *P* < 0.019]. Of the 70 ESCC patient tissues tested, 32 (45.7%) were defined as having high IGFBP-3 expression. The levels of IGFBP-3 protein expression were decreased in 70.0% (7 of 10) of ESCC tissues compared with adjacent non-malignant esophageal tissue. In addition, IGFBP-3 expression was associated with pathologic classification (*P* < 0.05 for T, N, and M categories and clinical stage). Patients with elevated protein level of IGFBP-3 in the tumor had an improved radiotherapy response and prolonged overall survival (*P* < 0.001).

**Conclusions:**

High level of IGFBP-3 expression in ESCC associates with early clinical stages and are predictive for favorable survival of the patients treated with radiotherapy.

## Background

Esophageal cancer is the leading cause of death from gastrointestinal malignancies worldwide, with an increasing incidence in Asian countries such as China [1]. The most prevalent histologic type of esophageal cancer in China is esophageal squamous cell carcinoma (ESCC) [1]. Due to the lack of reliable methods of early detection and absence of early symptoms, most patients with esophageal cancer are diagnosed with relatively advanced-stage disease. Despite recent advances in surgery and chemoradiotherapy, the prognosis of patients with esophageal cancer is very poor, with a 5-year survival rate of <30% [2]. Unfortunately, little progress has been achieved in improving long-term survival for several decades. Radiotherapy is a major component of treatment for locally advanced ESCCs. However, the radiosensitivity of individual tumors varies widely, and treatment failure in ESCC patients is partly due to radioresistance. This means that some patients are unresponsive to radiotherapy [3]. Therefore, various tumor markers are now employed to predict the degree of radiosensitivity, response to treatment, likelihood of relapse, prognosis of ESCC, as well as to develop targeted therapy [4–6]. However, several markers are regarded as having poor specificity and/or sensitivity. The role of tumor markers remains to be further defined.

Insulin-like growth factors (IGFs) are present throughout the body almost entirely in association with six specific high-affinity binding proteins, which play critical roles in the regulation of IGF action and availability [7]. Insulin-like growth factor-binding protein-3 (IGFBP-3) has a molecular mass of 28.7 kDa, and the mature protein comprises 264 amino acids. Serving as a major carrier protein for the IGF system, IGFBP-3 is known to modulate IGF/IGF type I receptor (IGF-IR)-dependent and -independent actions in the circulation and immediate extracellular environment [8]. In addition, IGFBP-3 has multiple functions in inhibiting cell proliferation and activating proapoptotic factors in various cell lines [8, 9]. It is present in the tumor tissues of most adult patients, and it is synthesized and secreted by various cell types in vitro [8]. According to early epidemiological studies, low levels of IGFBP-3 were independently associated with a high risk of human malignancies, such as colorectal cancer, lung cancer, and breast cancer [10–12]. This study aimed to evaluate whether IGFBP-3 plays a role in predicting the degree of radiosensitivity, response to treatment, and prognosis in ESCC patients.

## Patients and methods

### Patients and tissue specimens

Paraffin-embedded primary ESCC tissue samples from 70 consecutive ESCC patients treated with radiotherapy alone were obtained from Sun Yat-Sen University Cancer Center, Guangzhou, China between 2002 and 2011. The patients with available biopsy specimens and follow-up data were included; the patients whose cause of death remained unknown were excluded from the study. Ten pairs of fresh primary ESCC tissues and adjacent normal esophageal mucosa tissues were collected at the time of surgical resection in 2011. All of the samples used were endoscopic biopsy specimens obtained before treatment. The clinicopathologic characteristics of the tumor sets are shown in Table 1. Tumor stage was assessed according to the American Joint Committee on Cancer (AJCC) staging system (6th edition).

**Table 1:** Association of insulin-like growth factor-binding protein-3 (IGFBP-3) expression with clinicopathologic characteristics of ESCC patients

Variable	No. of patients	IGFBP-3 expression [cases (%)]		*P* value^a^
		High	Low	
Age^b^				0.069
>60 years	57	29 (50.9)	28 (49.1)	
≤60 years	13	3 (23.1)	10 (76.9)	
Sex				0.484
Male	45	20 (44.4)	25 (55.6)	
Female	25	12 (48.0)	13 (52.0)	
Location				0.722
Cervical	9	5 (55.6)	4 (44.4)	
Thoracic	61	27 (44.3)	34 (55.7)	
Tumor size^c^				0.585
>6 cm	22	9 (40.9)	13 (59.1)	
≤6 cm	48	23 (47.9)	25 (52.1)	
T category				0.003
T1–2	19	14 (73.7)	5 (26.3)	
T3	31	14 (45.2)	17 (54.8)	
T4	20	4 (20.0)	16 (80.0)	
N category				0.021
N0	21	14 (66.7)	7 (33.3)	
N1	49	18 (36.7)	31 (63.3)	
M category				0.006
M0	50	28 (56.0)	22 (44.0)	
M1-lym^d^	20	4 (20.0)	16 (80.0)	
Clinical stage				0.003
II	19	14 (73.7)	5 (26.3)	
III	31	14 (45.2)	17 (54.8)	
IV	20	4 (20.0)	16 (80.0)	
Survival status				0.01
Alive	20	14 (70.0)	6 (30.0)	
Dead	50	18 (36.0)	32 (64.0)	
Radiotherapy response				<0.001
Complete reaction	24	20 (83.3)	4 (16.7)	
No complete reaction	46	12 (26.1)	34 (73.9)	

^a^By χ^2^-Test

^b^Mean age

^c^Mean tumor size

^d^Distant lymph node metastasis

All of the samples were evaluated by two pathologists. Histology was determined according to the criteria of the World Health Organization. The Institute Research Ethics Committee of Sun Yat-sen University Cancer Center granted approval for this study.

### Radiotherapy

External beam radiotherapy was performed using 6- to 10-MV X-rays. All of the patients received three-dimensional conformal radiotherapy at a dose of 1.8–2.0 Gy per fraction, 5 times per week. The patients underwent radiotherapy for 4–6 weeks, receiving a total dose of 46–70 Gy. The primary gross tumor volume (GTV) and gross tumor volume of involved lymph nodes (GTV-N) were determined by computed tomography (CT). The conformal clinical target volume (CTV) included the GTV with a 3-cm margin in the craniocaudal direction and a 0.5-cm margin in the lateral and anteroposterior directions. The CTV of ESCCs involving the upper one-third of the esophagus encompassed the right and left supraclavicular regions. In patients with unilateral cervical lymph node metastasis, the contralateral supraclavicular fossa was included in the CTV for prophylactic purposes. The CTV for lymph nodes included the GTV-N without an additional margin. The planning target volume included the CTV with a 1.0-cm margin in the superior-inferior direction and a 0.5-cm margin in the lateral direction [13].

### Immunohistochemistry

Immunohistochemical analysis of IGFBP-3 was performed by using a standard two-step technique as described previously [14]. Briefly, nonspecific antibody binding was blocked with 10% normal rabbit serum for 20 min. Tissue sections were incubated with a 1:50 dilution of anti-IGFBP-3 polyclonal antibody (Santa Cruz Biotechnology, Dallas, TX, USA) for 1 h at 37°C in a moist chamber. Sections were then incubated with a 1:100 dilution of biotinylated rabbit anti-mouse immunoglobulin for 30 min at 37°C. The primary antibody was replaced with normal murine IgG in negative controls. Immuno-positive tissue sections were used as positive controls. Cytoplasm immunoreactivity for IGFBP-3 was scored by evaluating the number of positive tumor cells over the total number of tumor cells. Scores were assigned by a 5% increment from 0 to 100% and by two independent pathologists who were blinded to the clinical follow-up data.

### Selection of cut-off scores

Receiver operating characteristic (ROC) analysis was also performed with the protein marker to determine the cut-off scores for clinicopathologic features. Based on the IGFBP-3 score, the sensitivity and specificity for each outcome under study was plotted, thus creating an ROC curve. The score closest to the point with both maximum sensitivity and specificity (i.e., the point [0.0, 1.0] on the curve) was selected as the cut-off score. Low expression of the protein was defined as a score below or equal to the threshold value, and high expression was defined as a score above the threshold. To use ROC analysis, the clinicopathologic characteristics, T category, N category, M category, tumor grade, overall survival (OS), and progression-free survival (PFS) were assessed.

### Western blotting analysis

Total protein was isolated from 10 pairs of fresh endoscopic biopsy specimens of ESCC tissue and adjacent non-malignant esophageal tissue using Trizol buffer (Invitrogen, Carlsbad, CA, USA). Equal amounts of whole cell and tissue lysates were resolved by SDS–polyacrylamide gel electrophoresis (PAGE) and transferred onto polyvinylidene difluoride (PVDF) membranes. Blots were incubated with primary mouse monoclonal antibodies against human anti-IGFBP-3 (Santa Cruz Biotechnology, Dallas, TX, USA; 1:200 dilution), and immunoreactivity was detected using an enhanced chemiluminescence kit (Amersham Biosciences, Uppsala, Sweden). This procedure was performed as previously described [15]. All procedures were conducted in accordance with the manufacturer’s instructions.

### Statistical analyzes

Statistical analysis was applied using the SPSS statistical software package (standard version 13.0; SPSS, Chicago, IL, USA). The relationship between IGFBP-3 expression and ESCC patient clinicopathologic data was estimated using χ^2^ test. The association of survival with each variable was determined by using the log-rank test. Relative risks (RRs) of death associated with IGFBP-3 expression and other variables were estimated using univariate and multivariate Cox proportional hazards models. Differences were considered significant if the *P* value from a two-tailed test was <0.05.

## Results

### Selection of IGFBP-3 cut-off scores

**Fig. 1:**
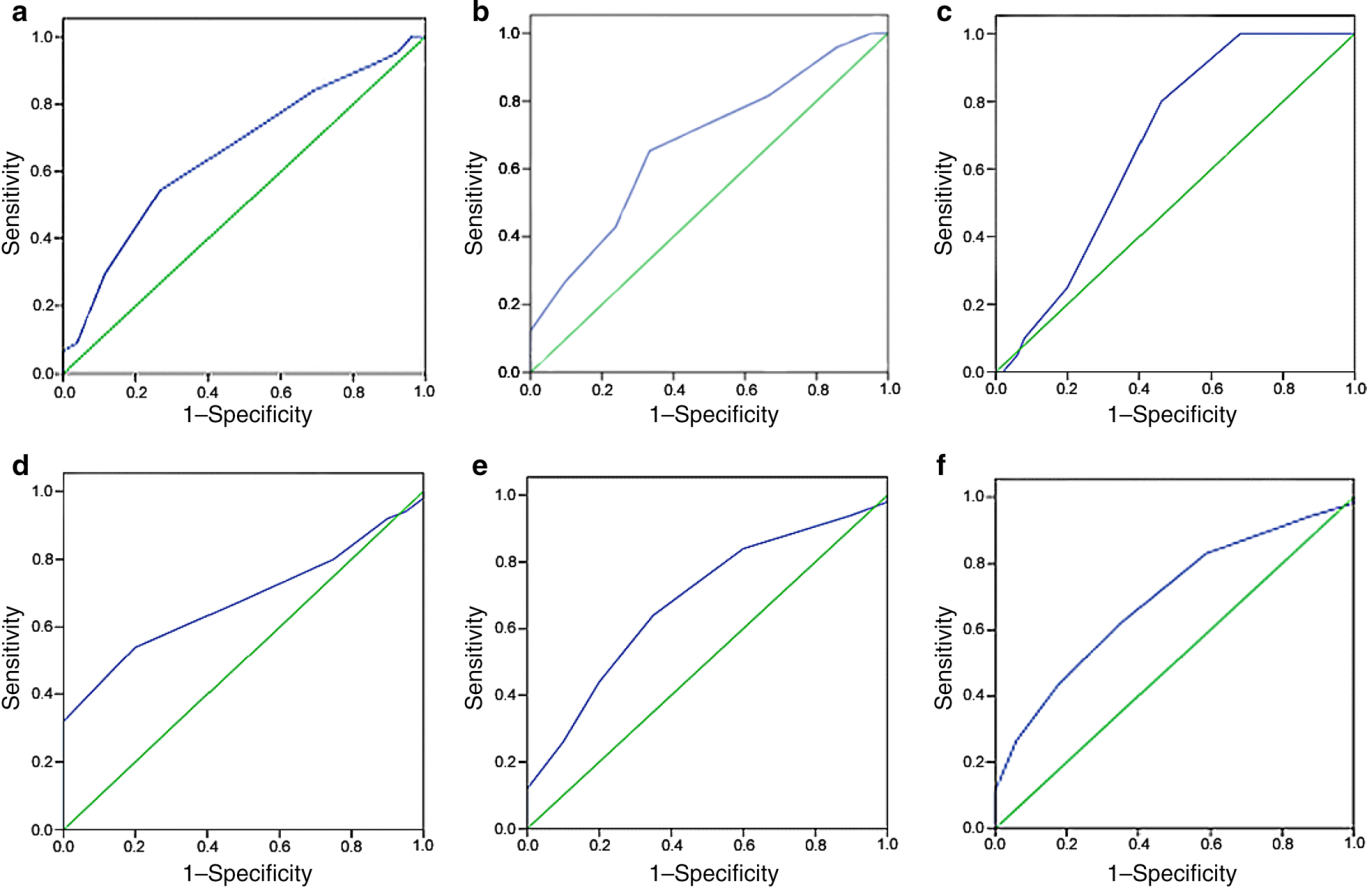
Receiver operating characteristic (ROC) analysis was performed to determine the cut-off score for the positive expression of insulin-like growth factor-binding protein-3 (IGFBP-3) in 70 esophageal squamous cell carcinoma (ESCC) patients. The sensitivity and specificity for each outcome were plotted: **a** T category, **b** N category, **c** M category, **d** tumor grade, **e** survival status, and **f** progression-free survival

The ROC analysis for each clinicopathologic parameter showed the point on the curve closest to (0.0, 1.0), which maximizes both the sensitivity and specificity for the outcome (Fig. 1). The corresponding areas under the ROC curve (AUC) with 95% confidence interval (CI) are shown in Table 2. According to the ROC analysis, values above the critical value of 0.65 were defined as positive for IGFBP-3 protein expression.

**Table 2:** Area under the receiver operating characteristic (ROC) curve (AUC) for each clinicopathologic feature of patients with esophageal squamous cell carcinoma (ESCC)

Feature	AUC (95% confidence interval)	*P* value^a^
T category	0.658 (0.553–0.828)	0.028
N category	0.674 (0.540–0.809)	0.021
M category	0.678 (0.555–0.800)	0.021
Clinical stage	0.678 (0.555–0.800)	0.021
Overall survival	0.683 (0.548–0.818)	0.017
Progression-free survival	0.690 (0.553–0.828)	0.019

^a^By χ^2^ test

### IGFBP-3 expression in ESCC tissues

**Fig. 2:**
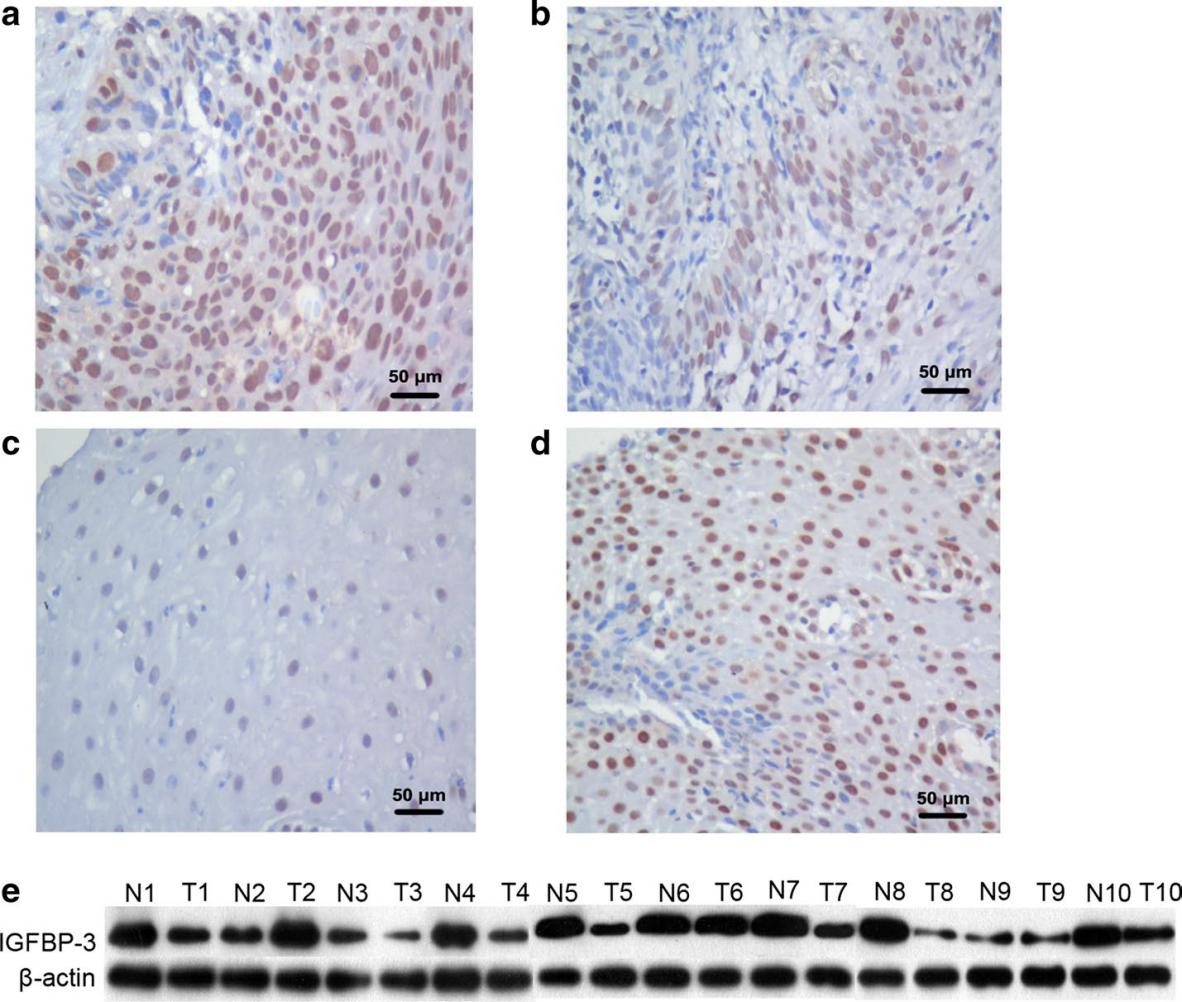
Immunohistochemical analysis of IGFBP-3 staining and western blotting analysis. **a** Strong IGFBP-3 staining in an ESCC case (case 16), in which approximately 80% of ESCC cells are stained positively for IGFBP-3 protein in the cytoplasm (×200). **b** Weak IGFBP-3 staining in an ESCC case (case 10) (×200). **c** Negative IGFBP-3 staining in a non-malignant esophageal mucosa specimen (×200). **d** Strong IGFBP-3 staining in a non-malignant esophageal mucosa specimen (×200). **e** 7 of 10 ESCC cases displayed low expression of IGFBP-3 by western blotting compared with adjacent non-malignant esophageal tissues (N1–10)

For IGFBP-3 immunohistochemical staining in ESCC tissues, immunoreactivity was observed as areas of yellowish-brown color primarily in the cytoplasm within tumor cells (Fig. 2). Immunoreactivity ranged from 0 to 100%. As shown in Fig. 2a, b, 45.7% (32 of 70) of ESCC cases were evaluated as having high IGFBP-3 expression, with the remaining ESCC cases (54.3%, 38 of 70) defined as having negative or low IGFBP-3 expression. IGFBP-3 was further examined by western blotting analysis in 10 pairs of fresh ESCC tissues and adjacent non-malignant esophageal specimens (Fig. 2c, d). The frequency of high IGFBP-3 expression was significantly lower in ESCC cases than in adjacent non-malignant esophageal tissues (70% [7 of 10] vs. 30% [3 of 10], *P* = 0.007) (Fig. 2e).

### Association of IGFBP-3 protein expression with clinicopathologic parameters

The expression rates of IGFBP-3 in ESCC with respect to several standard clinicopathologic features are listed in Table 1. No significant difference was observed between the IGFBP-3 expression level and clinicopathologic features such as patient age, sex, tumor location, and tumor size (*P* = 0.069, 0.484, 0.722 and 0.585, respectively, Table 1). However, the IGFBP-3 expression levels were found to be significantly higher in patients with earlier T category (*P* = 0.030), negative lymph node (*P* = 0.021), and no metastasis (*P* = 0.006).

### Association of IGFBP-3 protein expression in ESCC with patients’ radiotherapy response

Further analysis demonstrated that high IGFBP-3 expression was associated with an improved radiotherapy response in ESCC patients, with 83.3% of patients achieving complete response (CR) after radiotherapy for 3 months. Alternatively, low expression of IGFBP-3 was directly related to ESCC patients’ resistance to radiotherapy (*P* < 0.001, Table 1).

### Association between clinicopathologic variables as well as IGFBP-3 expression and ESCC patient survival

**Fig. 3:**
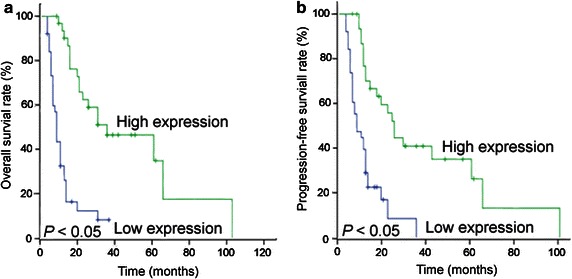
IGFBP-3 expression influences overall survival of ESCC patients. High expression of IGFBP-3 in 70 ESCC patients treated with radiotherapy alone was associated with prolonged **a** overall survival and **b** progression-free survival (*P* < 0.001; log-rank test)

Kaplan–Meier survival curves were confirmed by the log-rank test. The log-rank statistics showed a significant impact of well-known clinicopathologic prognostic parameters, such as sex (*P* = 0.036), tumor size (*P* = 0.028), T category (*P* < 0.001), N category (*P* = 0.003), and M category (*P* = 0.006), on patient survival (Table 3). In all cases, a high expression level of IGFPB-3 was found to be associated with improved OS and PFS (*P* < 0.001, Fig. 3). In addition, the median survival time of patients with high expression of IGFBP-3 was 25 months, whereas that of those with low expression of IGFBP-3 was 10 months (*P* < 0.001, Table 3).

**Table 3:** Clinicopathologic parameters and expression of IGFBP-3 for the prognosis of 70 ESCC patients by univariate survival analysis (log-rank test)

Variable	All cases	Mean survival (months)	Median survival (months)	*P* value^a^
Age^b^				0.739
>60 years	57	29.2	13.0	
≤60 years	13	19.0	18.0	
Sex				0.036
Male	45	19.4	13.0	
Female	25	46.4	24.0	
Location				0.316
Cervical	9	31.9	19.0	
Thoracic	61	26.8	13.0	
Tumor size^c^				0.028
>6 cm	22	14.3	13.0	
≤6 cm	48	33.7	20.0	
WHO grade				0.721
G1	9	28.4	19.0	
G2	29	28.0	12.0	
G3–4	32	24.0	18.0	
T category				<0.001
T1–3	50	37.0	17.0	
T4	20	16.7	12.0	
N category				0.003
N0	21	32.4	18.0	
N1	49	23.4	14.0	
M category				0.006
M0	50	33.9	19.0	
M1-lym^d^	20	15.1	12.0	
Clinical stage				
II	19	41.8	25.0	0.067
III	31	25.3	13.0	
IV	20	15.1	12.0	
IGFBP-3 expression				<0.001
High	32	44.6	25.0	
Low	38	10.9	10.0	

*WHO* World Health Organization

^a^By χ^2^ test

^b^Mean age

^c^Mean tumor size

^d^Distant lymph node metastasis

### Multivariate Cox regression analysis of independent prognostic factors of ESCC

The Cox proportional hazards model was applied to verify the independent value of each variable predicting OS. As shown in Table 3, the clinicopathologic features were a significant factor in predicting OS by univariate analysis. Multivariate analysis showed that the expression of IGFBP-3 was also a significant independent prognostic factor for predicting OS (*P* < 0.001; Table 4). The other variables investigated, including T category (*P* = 0.005), N category (*P* = 0.004), and M category (*P* = 0.003), had no significant effect on predicting OS.

**Table 4:** Multivariate analysis of overall survival (Cox regression model)

Variable	*β*	Relative risk	95% Confidence interval	*P* value^a^
Sex	0.433	0.649	0.32–1.31	0.229
Tumor size	0.206	0.814	0.399–1.659	0.82
IGFBP-3 expression	−1.328	0.265	0.130–0.541	<0.001
T category	0.933	0.393	0.206–0.750	0.005
N category	0.699	0.497	0.249–0.993	0.038
M category	0.787	0.455	0.222–0.935	0.032

^a^By χ^2^ test

## Discussion

The IGF system is well characterized, with profound effects on the proliferation and differentiation of normal and malignant cells. IGFBP-3 is a major carrier protein for IGFs. Growth inhibitory and proapoptotic effects of IGFBP-3 through both IGF-dependent and IGF-independent mechanisms have been well characterized [16]. Several studies have reported that IGFBP-3 induces apoptosis by reducing the bioavailability of IGF-1 to the IGF-1 receptor [17]. For instance, Alami et al. [18] found that recombinant human IGFBP-3 could inhibit the proliferation of lung cancer M-3 LL cells in a dose-dependent manner and could also significantly inhibit tumor growth in vivo. It was proposed that the *IGFBP*-*3* gene could be a putative tumor suppressor gene and/or therapeutic target for human cancers [19, 20]. Although the relationship between the *IGFBP*-*3* gene and human tumors has been investigated widely, the radiotherapy response and prognostic value of IGFBP-3 have not yet been established in ESCC.

In the present study, the expression of IGFBP-3 was assessed by immunohistochemistry in ESCC patients treated with radiotherapy alone and with clinicopathologic and follow-up data. IGFBP-3 immunoreactivity was assessed by a scoring system based on the percentage of positive tumor cells. This assessment method has been applied in colorectal cancer and adrenal cancer to evaluate the diagnostic or prognostic value of specific biomarkers [21]. ROC analysis was performed for each of the clinicopathologic parameters to set up more sensitive and specific immunohistochemistry cut-off scores for IGFBP-3 positivity. The cut-off score was ultimately determined to be above 0.65.

Immunohistochemistry revealed that 45.7% of the cases showed high cytoplasmic IGFBP-3 staining in ESCC tissue samples. In addition, western blotting analysis revealed down-regulated expression of IGFBP-3 in most ESCCs (70%) compared with their adjacent normal esophageal tissues (30%, *P* = 0.007). The IGFBP-3 expression level was significantly higher in patients with favorable prognostic factors, including earlier T category (*P* = 0.030), the absence of lymph node metastasis (*P* = 0.021), and distant metastasis (*P* = 0.006). Furthermore, high expression of IGFBP-3 was found to associate positively with improved radiotherapy response and to enhance radiosensitivity in ESCC patients. Univariate survival analysis showed that positive IGFBP-3 expression in ESCC was related to prolonged median survival time (25 vs. 10 months, *P* < 0.001). Moreover, high expression level of IGFBP-3 in ESCC was found to be an independent predictor of OS by Kaplan–Meier curves and multivariable Cox proportional hazards regression analysis. These results suggest that the *IGFBP*-*3* gene potentially facilitates apoptosis, inhibits tumor growth, and prevents cell invasion and/or metastasis in ESCC and that loss of IGFBP-3 expression may cause patients to have a poor prognosis.

These results are in accordance with the studies performed on malignant tumors that identified the tumor suppression action of IGFBP-3. Dar et al. [22] reported that overexpression of IGFBP-3 induces apoptosis and suppresses cell survival and growth in melanoma. Furthermore, there was evidence in this study that IGFBP-3 can induce apoptosis as well as potentiate the apoptotic effects of DNA damage induced by ionizing and ultraviolet irradiation. In a separate study, the effect of IGFBP-3 on the response of T47D cells to ionizing radiation was investigated, and the cells without IGFBP-3 expression appeared to be relatively radioresistant [23]. IGFBP-3 was transfected into T47D cells, causing an increase in radiosensitivity and IR-induced apoptosis by modulating the Bax and Bcl-2 protein ratio. These findings suggest that IGFBP-3 may be a potential predictor for radiosensitivity and could potentially offer a novel tool for radiotherapy response prediction and individualized therapy. Torng et al. [24] provided evidence indicating that IGFBP-3 also plays an important role as an invasion-metastasis suppressor in esophageal cancer, and low IGFBP-3 expression associated clinically with high tumor grade, advanced stage, and poor survival. In addition, down-regulation of IGFBP-3 in 86 gastric adenocarcinoma tissues relative to their adjacent non-cancerous tissues by immunohistochemistry was reported, and patients with high expression of IGFBP-3 showed a higher 5-year OS rate. Knock-down of *IGFBP*-*3* has also been shown to accelerate gastric cancer cell migration and invasion [25]. These findings led us to hypothesize that IGFBP-3 acts as a molecular prognostic marker in various cancers. To date, there has been no reference on radiotherapy response and prognostic significance of IGFBP-3 expression in ESCC. To our knowledge, this study shows for the first time the significance of the IGFBP-3 expression level on the response of ESCC patients to radiotherapy alone. In future studies, it would be desirable to provide insight into the potentially important role of IGFBP-3 as an underlying mechanism of development and radiosensitivity in ESCC.

## Conclusions

This study provides a basis for the concept that the positive expression of IGFBP-3 in ESCC may be important in the acquisition of radiosensitivity and an unaggressive prognostic phenotype. Loss of IGFBP-3, at least in part, accounted for the development and/or ultimately the progression of ESCC. Therefore, IGFBP-3 is a potential biomarker for predicting radiosensitivity and prognostic outcome in ESCC.
